# Effects of garlic polysaccharide on alcoholic liver fibrosis and intestinal microflora in mice

**DOI:** 10.1080/13880209.2018.1479868

**Published:** 2018-07-03

**Authors:** Yuchuan Wang, Min Guan, Xin Zhao, Xinli Li

**Affiliations:** a Department of Pediatrics, The Second Hospital of Dalian Medical University, Dalian, PR China;; b Department of Biotechonolgy, Dalian Medical University, Dalian, PR China

**Keywords:** Protein expression, dominant organisms, hepatoprotective activity

## Abstract

**Context:** Alcoholic liver fibrosis (ALF) is treatable and reversible consequence of liver disease. Intestinal microflora plays an important role in the progression of liver disease. Garlic (*Allium sativum* L. [Amaryllidaceae]) has been consumed as a traditional medicine to treat liver injury.

**Objective:** To investigate the effects of garlic polysaccharide (GP) on ALF and intestinal microflora in mice.

**Materials and methods:** KM mice were orally administered with alcohol (56%, 6 mL/kg) for 30 d to establish ALF model, and divided into four groups together with control group (water only). Hugan tablet (60 mg/kg) or GP (250 and 150 mg/kg) were given 5 h after each dose of alcohol. Biochemical markers in serum and liver homogenate were determined with kits. Alteration of intestinal microflora, and protein expressions of TGF-β1, TNF-α and decorin were detected.

**Results:** In GP-H group, ALT and AST decreased to 18.85 ± 4.71 U/L and 40.84 ± 7.89 U/L. MDA, TC, TG and LDL-C decreased to 2.32 ± 0.86 mmol/mg, 0.21 ± 0.12 mmol/L, 0.96 ± 0.31 mmol/L and 0.084 ± 0.027 mmol/L. SOD, GSH-Px and GSH increased to 118.32 ± 16.32 U/mg, 523.72 ± 64.20 U/mg and 0.56 ± 0.05 mg/g. Ratios of TGF-β1 and TNF-α decreased to 0.608 ± 0.170 and 1.057 ± 0.058, decorin increased to 2.182 ± 0.129. Lachnospiraceae and *Lactobacillus* increased, *Facklamia* and *Firmicutes* decreased with GP pretreatment.

**Discussion and conclusions:** Intestinal microflora provides novel insight into the mechanisms of GP that may be used to treat ALF and intestinal microflora dysbiosis.

## Introduction

Alcoholic liver disease (ALD) is a major cause of morbidity and mortality (Cho et al. 2017), and affects millions of individuals worldwide. The development of ALD involves some steps, which include alcoholic steatosis, alcoholic hepatitis, alcoholic liver fibrosis (ALF), alcoholic cirrhosis and liver cancer (Chuang et al. 2016). ALF develops from simple steatosis and hepatitis to cirrhosis and even liver cancer (Baghy et al. 2012). It is treatable and potentially reversible consequence of liver disease (Lee and Friedman 2010), and the effective antifibrotic methods significantly alter the management and prognosis of patients with liver disease (Friedman 2003). Thus, research focused on finding novel and effective treatment methods to reverse ALF is crucial.

Fibrotic process of the liver begins from lipid peroxidation and accumulation of extracellular matrix (ECM) which is principally produced by activated hepatic stellate cells (HSCs). Transforming growth factor-β1 (TGF-β1) increases the accumulation of these matrix proteins at the injury site and promotes fibrosis (Goetsch and Niesler 2016), whereas the proteoglycan decorin acts as an anti-fibrotic agent *via* the binding and neutralization of TGF-β1. Accumulating evidences demonstrated that ALF was prevented by reducing the expression of TGF-β1, tumour necrosis factor-α (TNF-α), and increasing the expression of decorin in the liver (Thu et al. 2016). Herbal medicines show potent effects against hepatic fibrosis (Liu et al. 2015) which have been used to treat ALF for a long time. So it is reasonable to develop new and effective natural products from medicinal herbs to treat ALF.

In recent years, several associations between common chronic human disorders and altered intestinal microflora composition and function have been reported (Forslund et al. 2015). Animals and humans exposed to alcohol chronically exhibit overgrowth of opportunistic pathogenic and depletion of beneficial intestinal bacteria (Cresci et al. 2017). Our previous study (Zhao et al. 2017) demonstrated that *Lactobacillus*, *Clostridium butyricum* and *Bacteroides* decreased in diabetic liver injury mice. Thus, there is a strong relationship between liver and gut. Alterations of intestinal microflora seem to play an important role in induction and furthering the progression of liver damage (Cesaro et al. 2011). Severe alcoholic hepatitis is associated with key changes to intestinal microflora, which influences individual sensitivity to develop advanced ALD. Intestinal microflora study should be considered as a new therapeutic target in ALD (Ferrere et al. 2017).

Garlic (*Allium sativum* L. [Amaryllidaceae]) has been consumed as a flavouring agent and a traditional medicine in China for many years to treat tuberculosis, coughs, colds, hyperpiesia, minor vascular disorders, diabetes, obesity, kidney and liver injury, and cancer (Naji et al. 2017). Organosulphur compounds and oil from garlic have attracted more attention (Pan and Wu 2014). However, little information is regarding the biological activity of garlic polysaccharide (GP). We therefore specifically used an ALF mice model to evaluate the effects of GP on the intestinal microflora, examine the mechanism of hepatoprotective activity of GP by the suppression of TNF-α, TGF-β1 and decorin, and try to explore the association between intestinal microflora and ALF.

## Materials and methods

### Plant material and reagents

Fresh garlic was purchased from a Dalian Lvshun local supermarket in August 2015 and identified by Professor Yuling Yin (Dalian Medical University) according to the standard of Pharmacopeia of the People’s Republic of China. A voucher specimen (No. GA 201501) is deposited in Department of Biotechnology, Dalian Medical University, China. Hugan tablet (Approval Number: Z22020994) was purchased from Changchun overseas Pharmaceutical Group Co., Ltd. (Changchun, China). Er Guotou white spirit was purchased from Beijing Red Star Co., Ltd. (Beijing, China). DEAE-52 cellulose was purchased from Whatman International Ltd. (Maidstone, Kent, UK). T-series dextrans (T-200, T-80, T-40, T-20 and T-10) were purchased from Phannacia (Piscataway, NJ). Stool DNA kit was purchased from ForeGenen (Chengdu, China). Polymerase Chain Reaction primers GC-357f (CGCCCGGGGCGCGCCCCGGGCGGGGCGGGGGACGGGGGGCCTACGGGAGGCAGCAG), 518r (ATTACCGCGGCTGCTGG) and 357f (CCTACGGGAGGCAGCAG) were synthesized by TaKaRa Biotechnology Co., Ltd. (Dalian, China). PCR Mix was purchased from Beijing TransGen Biotech Co., Ltd. (Beijing, China). Antibodies against TNF-α, TGF-β1, decorin, β-actin and HRP-conjugated affinipure goat anti-rabbit IgG (H + L) were obtained from Proteintech Group Inc. (Chicago, IL). The enhanced chemiluminescence (ECL) kit was from Amersham Life Science, Inc. (Arlington Heights, IL). Alanine aminotransferase (ALT), aspartate aminotransferase (AST), malondialdehyde (MDA), glutathione peroxidase (GSH-Px), glutathione (GSH), superoxide dismutase (SOD), total cholesterol (TC), triglyceride (TG), high density lipoprotein cholesterol (HDL-C) and low density lipoprotein cholesterol (LDL-C) were purchased from the Jiancheng Bioengineering Institute (Nanjing, China). All other chemical reagents used were analytical grade.

### Isolation and purification of GP

GP was prepared according to the previous report with slight modifications. Briefly, the air-dried garlic (200 g) was crushed and extracted by hot reflux method three times with distilled water (800 mL) at 70 °C, 2.5 h for each time. The whole extract (300 mL) was treated with ethanol (700 mL) to a final concentration of 70% at 4 °C overnight. The precipitate was crude polysaccharide fraction. The crude polysaccharide solution (400 mL) was graded precipitated with 50% ethanol (600 mL), 70% ethanol (600 mL) and deproteinated by Sevag method (Staub 1965) to get purified crude polysaccharide (CGP, 5.31 g). The CGP solution was fractionated on DEAE-52 cellulose column. After elusion with water and gradient solutions (0.1 mol/L NaCl, 1.5 L and 0.5 mol/L NaOH, 1.5 L), the major polysaccharide fraction (GP) was evaporated under reduced pressure at 60 °C to dryness, and the produced dry powders were stored at 4 °C for subsequent experiments.

### Physicochemical property of GP

Total carbohydrate content of GP was determined by phenol-sulphuric acid colorimetric method. Protein content was quantified according to Bradford’s method. The purity of GP was evaluated by HPLC System (Waters E2695, Milford, MA) with an Acchrom Xamide column (4.6 × 150 mm, 5 μm; Acchrom, Beijing, China) and a RID-10A Refractive Index Detector. HPLC was performed on 0.5% GP (20 µL) dissolved in distilled water with MeOH–water (20:80, *v/v*) as the mobile phase at 0.6 mL/min and 35 °C. The molecular weight of GP was evaluated by HPLC equipped with a TSK gel G3000 PWXL column (7.8 × 300 mm) and a RID-10A Refractive Index Detector. HPLC was performed on 0.5% GP (20 µL) dissolved in distilled water with 0.7% Na_2_SO_4_ as the mobile phase at 0.5 mL/min and 35 °C. The columns were calibrated with T-series dextran as standards.

### Animals and experimental design

Male KM mice weighing 30 ± 3 g provided by the Experimental Animal Center of Dalian Medical University, Dalian, China (Quality certificate number: SCXK (Liao) 2013-0003). All experimental procedures were approved by the Animal Care and Use Committee of Dalian Medical University and performed in strict accordance with the People’s Republic of China Legislation Regarding the Use and Care of Laboratory Animals (Approval number: SYXK (Liao) 2013-0006; 18 November 2013). The mice were kept under standardized conditions at a temperature of 22–24 °C, and 20% humidity with a 12 h light/dark cycle, and they had free access to standard diet and water *ad libitum*. After acclimatization for one week, the animals were randomly divided into five groups (*n* = 10) (Guo et al. 2017): Normal control group: mice were orally administered distilled water only; Negative group: mice were orally administered with alcohol (Er Guotou white spirit, 56%, 6 mL/kg); Positive group: mice were orally administered with alcohol (56%, 6 mL/kg) and 5 h after each dose of alcohol with Hugan tablet (60 mg/kg); Group GP-H and GP-L: mice were orally administered with alcohol (56%, 6 mL/kg) and 5 h after each dose of alcohol with GP at doses of 250 and 150 mg/kg (Nasr 2017), respectively. All animals were administered for 30 consecutive days. After 16 h fasting following the last administration, the blood samples were collected from the eyeballs and centrifuged at 3000 rpm for 10 min at 4 °C to afford the serums. The faecal samples were collected and stored at −80 °C for intestinal microflora analysis. The liver samples were dissected out immediately after the mice were killed, and wash instantly with ice-cold physiological saline (0.9% NaCl solution), check the liver weight to calculate relative liver weight (relative liver weight (%) = liver weight/body weight × 100), and one part of liver tissue was rapidly divided and fixed in 10% formalin for pathological examination, and the remaining parts were stored at −80 °C for the liver biochemical assays and western blotting assay.

### Biochemical analysis

The liver tissue was minced, homogenized in ice-cold physiological saline by a glass homogenizer, and centrifuged at 3000 rpm for 10 min at 4 °C to afford the 10% (w/v) liver homogenate. The levels of ALT and AST in the serum as well as the levels of MDA, GSH-Px, GSH, SOD, TC, TG, HDL-C and LDL-C in the liver homogenate were determined using commercial kits. All of the procedures were carried out according to the manufacturers’ instructions.

### Liver histopathology

Liver tissues were cut into 3 mm thick slices and fixed in 10% formalin for 24 h, paraffin embedded, sliced into 5 mm sections, and stained with hematoxylin-eosin (H&E) for histopathological examination. Each sample was observed at 100× magnification.

### Western blotting assay

Total protein was extracted from the liver samples using RIPA lysis buffer with protease inhibitors in a proportion of 1:100. The BCA assay kit was used to quantitate protein. Equal amounts of protein (100 µg) were separated by 10% SDS-PAGE gel using 100 V for 3 h and then transferred to nitrocellulose membrane by semi-dry apparatus for 50 min for β-actin, 25 min for TNF-α, 30 min for TGF-β1 and 50 min for decorin, respectively. The membranes were blocked with 5% nonfat milk for 2 h at room temperature and then incubated with primary antibodies against TNF-α, TGF-β1, decorin and β-actin, respectively, at a 1:500 dilution overnight at 4 °C. The next day, the membranes were incubated with secondary antibody at a 1:2000 dilution for 2 h at room temperature after washed with TBST for three times. Then, the protein bands were visualized using ECL kit by Bio-rad ChemiDoc XRS plus image analyzer (Bio-Rad, Hercules, CA) after TBST washing as previously described. β-actin was used as internal reference.

### Deoxyribonucleic acid (DNA) extraction

DNA was extracted from faecal samples with Stool DNA kit in accordance with the manufacturer’s instructions.

### Polymerase chain reaction (PCR) amplification

Primers GC-357f and 518r were used to amplify the V3 region of bacterial 16S rRNA. PCR amplification was performed with the methods reported in our previous study (Li et al. 2013).

### DGGE analysis

The PCR products were electrophoresed on 8% polyacrylamide (acrylamide/bisacrylamide, 37.5:1) gels containing a linear denaturant gradient ranging from 25 to 50%, with 100% denaturant defined as a solution of 7 M urea and 40% (*v/v*) deionized formamide. Electrophoresis was performed, first for 10 min at 200 V, and subsequently for 16 h at 70 V in a 1 × TAE buffer at a constant temperature of 60 °C. Gels were stained with ethidium bromide.

### Sequence analysis

Some separated and strong bands were cut out and eluted in 20 μL sterile water at 4 °C overnight. The eluted DNA was reamplified using 357f and 518r primers with the same PCR program. Subsequently, idiographic sequences were attained by TaKaRa Biotechnology (Dalian) Co. Ltd. (Dalian, China). Finally, the sequences were compared directly with those in GeneBank by Blast search (http://blast.ncbi.nlm.nih.gov/Blast.cgi).

### Statistical analysis

The statistical software SPSS version 17.0 (SPSS Inc., Chicago, IL) was used for analysis. *p* Values were determined using the Student’s *t*-test, *p* value < 0.01 was considered significant. DGGE and Western blotting gels were analysed by using Quantity One 4.6.2 gel analysis software (Bio-Rad, Hercules, CA). Similarities were displayed graphically as a dendrogram. The clustering algorithms used to calculate the dendrograms were an unweighted pair group method with arithmetic average (UPGMA). The Shannon-Wiener index of diversity (*H*′) was used to determine the diversity of the bacterial community. The evenness (E) which reflected uniformity of bacterial species distribution was also computed.

## Results

### Isolation and purification of GP

**Figure 1.**
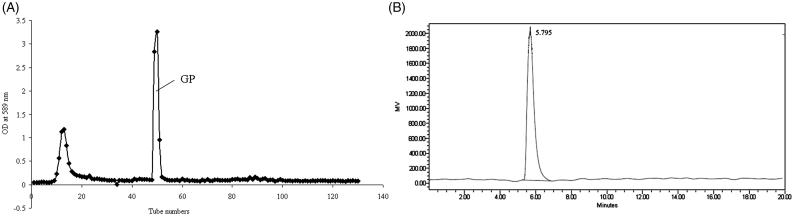
Elution curve of crude polysaccharide on DEAE-52 cellulose chromatography (A) and HPLC profile of GP (B).

The crude polysaccharide from garlic was extracted by hot water and ethanol precipitation with a yield of 2.7%. After deproteinated by a combination of proteinase and Sevag method, the crude polysaccharide sample was purified by DEAE-52 Cellulose to obtain GP with a yield of 1.76% (Figure 1(A)).

### Physicochemical properties and chemical compositions

GP appeared as a white powder, and had a negative response to Bradford assay. No absorption at either 260 or 280 nm was detected by UV spectrophotometer. These results showed the absence of nucleic acid and protein in GP. HPLC profile indicated that GP had a single and symmetrically sharp peak (Figure 1(B)), revealing that GP was a homogeneous polysaccharide with a purity of 98% by area normalization method. Phenol-sulphuric acid assay showed GP contained 87.2% carbohydrate. The molecular weight of GP was 10 kDa, and it is an acid heteropolysaccharide which consists of Fru (fructose), Gal (galactose) and Gal-A (galacturonic acid) in the ratio of 307:25:32 (Li et al. 2017).

### Effects of GP on serum and hepatic biochemistry

**Figure 2.**
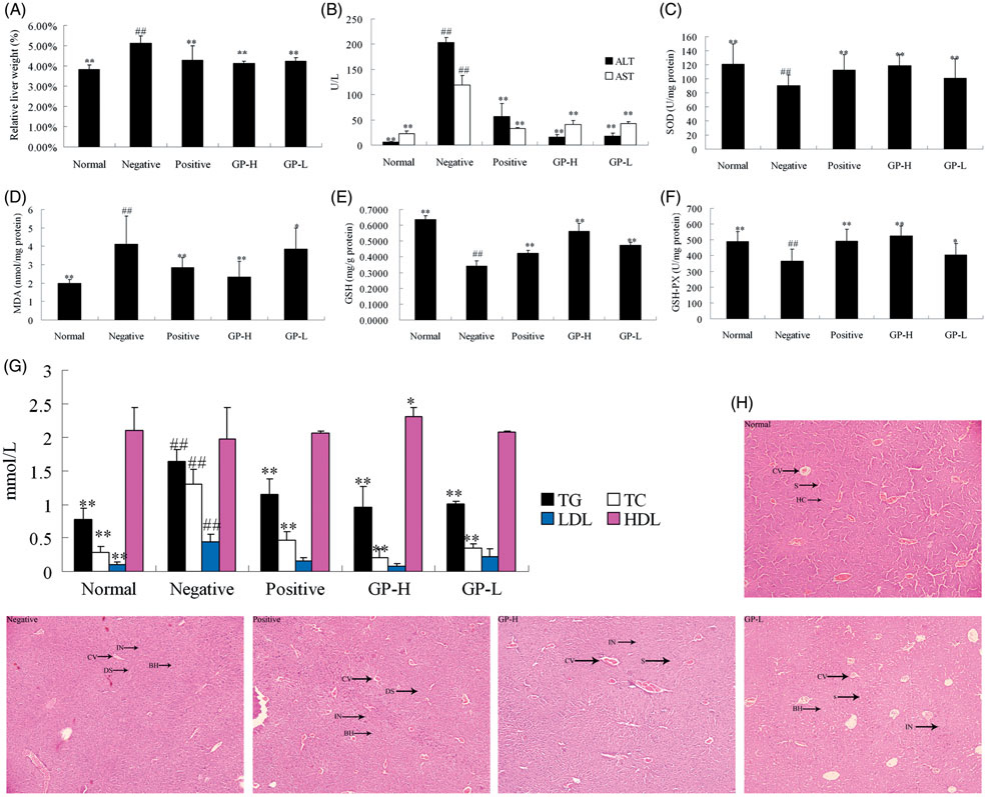
Effects of GP on the relative liver weight (%) (A), the levels of ALT and AST in serum (B), the activity of SOD in liver homogenate (C), the level of MDA in liver homogenate (D), the level of GSH in liver homogenate (E), the activity of GSH-Px in liver homogenate (F), the levels of TC, TG, HDL and LDL in liver homogenate (G), and H&E staining for histopathological examination (100×) (H). GP-H: GP-treated (250 mg/kg) group; GP-L: GP-treated (150 mg/kg) group. Values are expressed as mean ± SD (*n* = 10). *p < 0.05 and **p < 0.01 versus negative group; ##*p* < 0.01 versus normal control group. HC: hepatocytes; CV: central vein; S: sinusoid; DS: dilated sinusoid; IN: cellular inflammation; BH: ballooned hepatocytes.

As shown in Figure 2(B), AST and ALT levels elevated significantly (*p* < 0.01) in negative group indicated the increased permeability and damage of liver with alcohol administration. GP at the dose of 250 and 150 mg/kg significantly prevented alcohol-induced elevations of ALT (*p* < 0.01) and AST (*p* < 0.01) levels, which were equivalent to the effects produced by Hugan tablet at the dose of 60 mg/kg.

Levels of MDA, GSH, TC, TG, HDL, LDL and the activity of SOD, GSH-Px (shown in Figure 2(C–G) were monitored to evaluate the effects of GP on alcohol-induced liver lipid peroxidation and oxidative stress. Hepatic MDA, TC, TG and LDL levels were significantly (*p* < 0.01) increased, while SOD, GSH-Px activities and GSH level in liver homogenates were significantly (*p* < 0.01) decreased in negative group. However, the liver index (Figure 2(A)) of the mice pretreated with GP was considerably ameliorated. Moreover, MDA, TC, TG and LDL levels were significantly reduced, while SOD, GSH-Px activities and GSH level were markedly increased in GP-pretreated groups. The effects on MDA, SOD, GSH, GSH-Px, TC, TG, HDL, LDL and liver index in mice produced by GP at the dose of 250 mg/kg were equivalent or even better than the actions produced by Hugan tablet (positive drug). These results indicate that GP has a potential hepatoprotective activity.

### Effects of GP on histopathological changes

As shown in Figure 2(H), normal liver lobular architecture and cell structure were observed in normal control group. While the liver tissue of the mice in negative group showed apparent morphological changes. The presence of some areas of interstitial edema parallel to the cords of hepatocytes and the lobular structures were disappeared. Furthermore, massive inflammatory cells infiltration and ballooned hepatocytes were observed in the injured area. However, GP pretreatment significantly decreased the injured area, necrotic cells, inflammatory infiltration and ballooned hepatocytes.

### The expression of TNF-α, TGF-β1 and decorin by GP pretreatment

**Figure 3.**
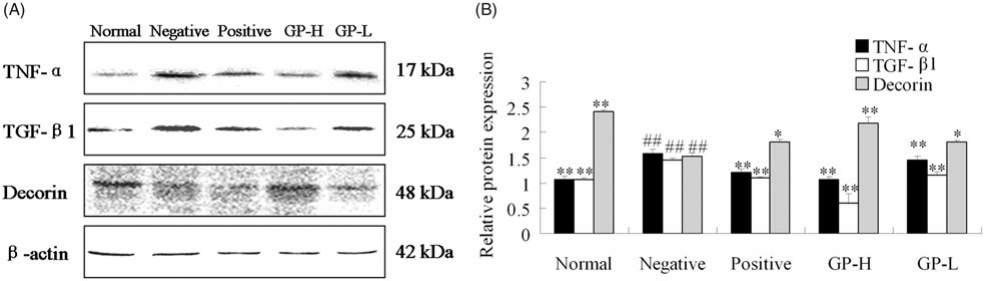
Effects of GP on protein expression of TGF-β1, TNF-α and decorin in livers. GP-H: GP-treated (250 mg/kg) group; GP-L: GP-treated (150 mg/kg) group. Values are expressed as mean ± SD (*n* = 10). *p < 0.05 and **p < 0.01 versus negative group; ##*p* < 0.01 versus normal control group.

Western blotting analysis (Figure 3) showed that the expression levels of TGF-β1 and TNF-α were significantly increased in the mice chronically administered with alcohol (*p* < 0.01). Meanwhile, the expression level of decorin was reduced with statistical significance (*p* < 0.01) in this group. Hugan tablet and GP pretreatment could dramatically decrease TGF-β1 and TNF-α expression levels (*p* < 0.01). In contrast, the expression of decorin increased as compared to negative group with statistical significance (*p* < 0.05). It suggested that the hepatoprotective activity of GP on ALF was at least partially related to inhibit the expression of TGF-β1, TNF-α and promote the expression of decorin.

### DGGE analysis

**Figure 4.**
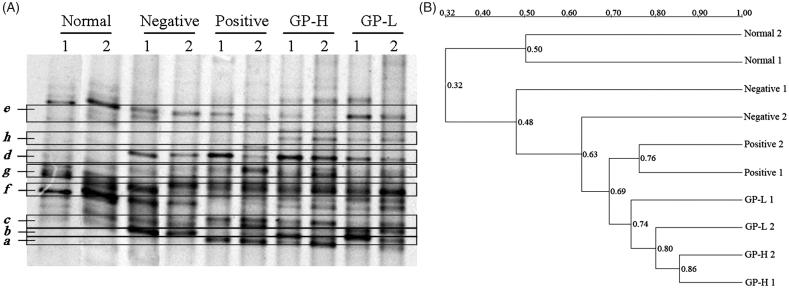
Representative DGGE profiles (A) and UPGMA dendrograms (B) of intestinal microflora. GP-H: GP-treated (250 mg/kg) group; GP-L: GP-treated (150 mg/kg) group.

The dominant intestinal microflora was examined by DGGE analysis with universal primers targeting the V3 region of the 16S rRNA (Figure 4(A)). Obviously, bands *b*, *c* and *d* were found in ALF groups, but they did not exist in the normal control group. Especially the intensities of bands *b* and *c* were highest in negative group, and the intensity of band *d* was high in positive and GP-pretreated (250 mg/kg) groups. Bands *e*, *f* and *g* existed in normal control group, but almost disappeared in other four groups. Band *a* increased remarkably in positive and GP-pretreated (250 mg/kg) groups. Band *h* only existed in GP-pretreated (250 and 150 mg/kg) mice groups. Other bands almost existed in all groups, and in the different groups, the intensity of the bands which in the same position was similar.

The clustering analysis based on the values of Dice coefficients was visualized in an UPGMA dendrogram to study general patterns of community similarity among the different groups by Quantity One software. Figure 4(B) displays that different groups formed the statistical significant clustering profiles. There were two main clusters in the dendrogram, the first was normal-1 and normal-2 related normal control group, the second was remaining four groups. The minimum bacterial similarity index between cluster one and cluster two was 0.32, which suggested that the intestinal microflora community of ALF mice was damaged seriously. In cluster two, similarity among positive, GP-H and GP-L groups was high (0.69), which suggested that the effects of Hugan tablet and GP (250 and 150 mg/kg) on intestinal microflora community were similar. In addition, similarity between negative group and other groups was 0.48, which suggested that the intestinal microflora community of Hugan tablet and GP pretreated mice was different from that of the mice administered with alcohol only.

DGGE profiles displayed the typical characteristics of general bacteria in the intestinal tract. Each band derives possibly from one phylogenetically distinct community; hence, an estimation of species number could be based on the total number of the bands in the profile. The Shannon-Wiener indexes of *H*′ reflecting the structural diversity of the bacterial community were calculated on the basis of the number and relative intensities of bands on the gel (Table 1).

**Table 1.** Microflora diversity indexes analysis^a^.

Group	S	*H*’^b^	E^c^
Normal	9.75 ± 0.96*	2.0371 ± 0.0443	0.9271 ± 0.0241
Negative	8.25 ± 1.50#	1.8988 ± 0.1624#	0.8562 ± 0.0101
Positive	9.75 ± 1.50	2.0409 ± 0.0931	0.9543 ± 0.0231
GP-H	13.5 ± 0.58**	2.4638 ± 0.0125**	0.9606 ± 0.0207
GP-L	12.75 ± 0.96**	2.4144 ± 0.0496**	0.9429 ± 0.0112

GP-H: GP-treated (250 mg/kg) group; GP-L: GP-treated (150 mg/kg) group.

Results are expressed as mean ± SD (*n* = 10). ^a^*p < 0.05 and **p < 0.01 versus negative group; #*p* < 0.01 versus normal control group. ^b^*H*’ = −∑ (*p_i_*) (ln*p_i_*), *p_i_* was the proportion of the bands in the track, *p_i_*= *n_i_*/∑*n_i_*, *n_i_* was the average density of peak *i* in the densitometric curve. ^c^E=*H*’/ln S, S was the number of bands.

It was clearly shown that diversity in negative group decreased significantly (*p* < 0.05) as compared to normal control group, while that in GP-H and GP-L groups increased significantly (*p* < 0.01). Compared to normal control group, the number of bands was lower in negative group, but richer in GP-H and GP-L groups, they all showed significant (*p* < 0.05) differences. Positive, GP-H and GP-L groups produced high evenness score, but negative group showed relatively low score, apparently due to a richer diversity of intense bands in the above three groups. Based on these results, it appeared that intestinal microflora community was changed by alcohol seriously, the richness (S), diversity index (*H*′) and evenness score (E) decreased. However, the intestinal microflora community of the mice pretreated with positive drug and GP was considerably ameliorated. The richness (S), diversity index (*H*′) and evenness score (E) increased markedly.

Data in Table 2 show the closest relatives based on results of BLAST searches with DNA sequences obtained from DGGE gel bands identified by cluster analysis. Bands in the same position but in different lanes were excised and sequenced to confirm that they had the same identity (data not shown). *e*, *f* and *h* were sequenced and identified as *Lachnospiraceae bacterium* with the similarity of 96, 87 and 98%, respectively. *a* was sequenced and identified as *Prevotella* sp. with the similarity of 95%. *d* was sequenced and identified as *Lactobacillus acidophilus* with the similarity of 98%. Lachnospiraceae existed in normal group, as well as the intensity weakened with alcohol administration. Interesting that one of Lachnospiraceae (*h*) was dramatically increased in GP-pretreated groups. *Lactobacillus* and *Prevotella* both existed in Hugan tablet and GP-pretreated (250 mg/kg) groups, while there was nearly no band at the corresponding place from normal control group particularly. *Lactobacillus* although existed in negative group, but the intensity was weaker than that in positive, GP-H and GP-L groups. *b* was sequenced and identified as *Facklamia ignava* with the similarity of 96%. *c* was sequenced and identified as *Firmicutes bacterium* with the similarity of 92%. They both existed in negative, positive, GP-H and GP-L groups, and increased remarkably in negative group. *g* was sequenced and identified as *Psychrobacter* sp. with the similarity of 99%.

**Table 2.** Sequences of PCR amplicons derived from DGGE gels and identities based on the BLAST database.

Selected band	Most similar sequence relative (GenBank accession number)	Bacteria genus	Identity (%)
*g*	*Psychrobacter* sp. (NZ AYXN01000033.1)	*Psychrobacter*	99
*d*	*Lactobacillus acidophilus* (NC 006814.3)	*Lactobacillus*	98
*c*	*Firmicutes bacterium* (NZ KE159700.1)	*Firmicutes*	92
*b*	*Facklamia ignava* (NZ JH932300.1)	*Facklamia*	96
*f*	*Lachnospiraceae bacterium* (NZ JH590865.1)	*Lachnospiraceae*	96
*e*	*Lachnospiraceae bacterium* (NZ JH590864.1)		87
*h*	*Lachnospiraceae bacterium* (NZ KE159593.1)		98
*a*	*Prevotella* sp. (NZ LFQU01000071.1)	*Prevotella*	95

DGGE analysis indicated that the intestinal microflora of mice was changed with alcohol administration obviously. The bacterium from the genera of Lachnospiraceae, *Lactobacillus*, *Prevotella*, *Facklamia*, *Firmicutes* and *Psychrobacter* were dominant organisms in the intestinal tract of mice of different groups by DGGE analysis. We further proposed that the alterations of dominant intestinal microflora play a causal role in ALF. Specifically, Lachnospiraceae and *Lactobacillus* decreased, while *Facklamia* and *Firmicutes* increased in alcoholic groups, which showed that the intestinal microflora balance was disturbed by alcohol, but GP pretreatment could mitigate intestinal microflora dysbiosis notably, Lachnospiraceae and *Lactobacillus* increased, while *Facklamia* and *Firmicutes* decreased with GP pretreatment.

## Discussion and conclusions

Under ALF, the quiescent HSCs trans-differentiate into fibrogenic myofibroblast-like cells caused by platelet-derived growth factor (PDGF), TNF-α, TGF-β1 or reactive oxygen species (ROS) (Dong et al. 2015), with expression of α-smooth muscle actin and type I collagen, and secreting profibrogenic mediators, ultimately promoting the progression of liver fibrosis (Bai et al. 2016). In the matrix overproduction, TGF-β1 occupies a central position. It has been considered as the most important profibrogenic cytokine, which contributes to the development of liver fibrosis through modulating the synthesis and degradation of ECM proteins (Zhang et al. 2015). Blockade of TGF-β1 activity has proven to be an effective way of inhibiting the fibrotic response to injury in various organs (Baghy et al. 2012). In addition, decorin is a small ECM dermatan sulphate-chondroitin sulphate proteoglycan that is present in low quantities in the normal liver. It is known to act as an anti-fibrotic agent *via* the binding and neutralization of TGF-β1 (Goetsch and Niesler 2016). Thus, enhanced deposition of decorin could reflect the stimulatory effect of overproduced TGF-β1, without necessarily exerting a protective role against fibrosis.

Development of novel and effective treatment method to reverse ALF is critical (Safadi and Friedman 2002). In this work, GP showed significant effects against ALF as evidenced by the decreased AST and ALT levels as well as the relative liver weight, decreased liver MDA, TC, TG and LDL levels, increased SOD, GSH-Px activities and GSH level, and alleviated histopathological changes. Furthermore, GP was effective in reducing the expression of TGF-β1, TNF-α, and promoting the expression of decorin to inhibit HSCs activation and reduce ECM accumulation for attenuating liver fibrosis.

Intestinal microflora participates to the metabolism of alcohol. The metabolites increase intestinal permeability indirectly through changing the microbiota equilibrium. And the increased the bacterial lipopolysaccharide arrives at the liver (Visapää et al. 2002) which induces the liver injury. The intestinal microflora in people with alcoholism demonstrates significant difference in respect to the microflora of a control group (Bode et al. 1984). Probiotics/prebiotics have been suggested as a useful integrative treatment of chronic liver damage, for their ability to improve intestinal barrier function and prevent bacterial translocation. For example, patients treated with probiotics had a restoration of intestinal microflora with an increased number of both *Bifidobacteria* and *Lactobacilli* (Kirpich et al. 2008). Lactitol treatment could decrease endotoxemia through an increase in *Bifidobacterium* and *Lactobacillus* and an inhibition of potentially pathogenic bacteria growth (Chen et al. 2007). Supplementation with probiotics/prebiotics is associated with greater improvement in alcohol-induced liver injury than standard therapy. Therefore, probiotic/prebiotic treatment in alcohol-related liver disease is more important. In this work, Beneficial populations such as Lachnospiraceae and *Lactobacillus* decreased, whereas potentially pathogenic bacteria such as *Facklamia* and *Firmicutes* increased in alcoholic groups, which showed that the intestinal microflora balance was disturbed by alcohol, and GP pretreatment mitigated intestinal microflora dysbiosis notably. As reported, bacterial phylotypes decrease in patients with cirrhosis was mostly associated with Lachnospiraceae (Chen et al. 2011). Lachnospiraceae is known as participating in carbohydrate fermentation into short-chain fatty acids (SCFAs) in the intestine. Lachnospiraceae suppression causes a decline in SCFAs production, which further results in increased colonic pH and increased ammonia production and absorption in the gut. Our findings show that Lachnospiraceae plays a vital role in ALF. *Lactobacillus* is antagonistic to pathogenic microorganisms of intestinal microflora, which help host to decompose polysaccharide and improve the efficiency of nutrition, increase lymphocyte numbers, speed up the vascularization of the gut mucosa, and modulate the gastrointestinal microflora (Wang et al. 2016). *Facklamia* is an emerging pathogen that may be responsible for opportunistic infections in humans (Abat et al. 2016). To date, all the *Facklamia* spp. have been isolated from human clinical specimens with the exception of *F. tabacinasalis* and *F. miroungae*, which were isolated from tobacco powder and from an elephant seal nasal swab, respectively (Corona et al. 2014). Our data first show that *Facklamia ignava* exists in the gut of rodents. In addition, there is a strong relationship between *Bacteroidetes*/*Firmicutes* and obesity. Compared to lean mice, genetically obese mice have 50% fewer *Bacteroidetes*, and a proportional increase in *Firmicutes* (Ley et al. 2006). Taken together, the prevalence of potentially pathogenic bacteria such as *Facklamia* and *Firmicutes* with the decrease of beneficial populations such as Lachnospiraceae and *Lactobacillus* might contribute to the interaction between the intestinal microflora and ALF. Alcohol-induced modification of the intestinal microflora leads to the development of ALF. Intestinal microflora dysbiosis is associated with alcohol intake and a reversal of liver injury occurred by GP pretreatment. GP has prebiotic-like effects which can induce microbial competition and reduce the populations of non-beneficial intestinal microflora. Further studies are required to decipher the role of the intestinal microflora in the development of ALF and the effect of prebiotics (GP and so on) on the intestinal microflora.

In conclusion, GP has a significant hepatoprotective effect against ALF in mice through modulating lipid peroxidation and oxidative stress, regulating TGF-β1, TNF-α and decorin signalling pathways to restrain HSCs activation and decrease ECM production. Notably, the underlying mechanisms are certainly more complex than what is described here. In addition, the intestinal microflora alterations provide novel insights into the mechanisms of GP as a potent anti-fibrotic agent and prebiotic that may be used to treat ALF and ALF-induced intestinal microflora dysbiosis.
